# Supplementary material for: In Vitro and in Field Response of Different Fungicides against Aspergillus flavus and Fusarium Species Causing Ear Rot Disease of Maize
Source: Toxins (Basel). 2019 Jan 1;11(1):11. doi: 10.3390/toxins11010011 (PMC6357132; doi:10.3390/toxins11010011)
Supplement: Supplementary file 1 [file toxins-11-00011-s001.pdf]

Supplementary Materials: In Vitro and in Field Response of Different Fungicides against *Aspergillus flavus* and *Fusarium* Species Causing Ear Rot Disease of Maize

Mario Masiello, Stefania Somma, Veronica Ghionna, Antonio Francesco Logrieco and Antonio Moretti

Table S1. Colony growth inhibition on PDA amended with three different concentrations of active ingredient (A.i.) for each fungicide, after 3 days of incubation at 25 °C. The standard error ranged between 0 and 7.5.

Tested molecules	A.i concentration (mg L^{-1})	*F. graminearum*			*F. proliferatum*			*F. verticillioides*			*A. flavus*		
		126	***6352***	***6415***	***12072***	***12103***	***16031***	***12052***	***12043***	***12044***	***8111***	***8115***	***8095***
Prothioconazole	2	100	100	100	100	100	100	100	100	100	100	100	100
	20	100	100	100	100	100	100	100	100	100	100	100	100
	200	100	100	100	100	100	100	100	100	100	100	100	100
Prochloraz	4	100	100	100	100	100	100	100	100	100	100	100	100
	40	100	100	100	100	100	100	100	100	100	100	100	100
	400	100	100	100	100	100	100	100	100	100	100	100	100
Metconazole	0.9	94 ± 1.1	100	100	100	93 ± 0.0	100	100	100	100	69 ± 0.0	64 ± 2.3	59 ± 1.3
	9	100	100	100	100	100	100	100	100	100	100	100	100
	90	100	100	100	100	100	100	100	100	100	100	100	100
Propiconazole	2.5	73 ± 2.0	70 ± 1.2	75 ± 2.3	58 ± 4.2	71 ± 2.2	72 ± 1.4	90 ± 1.0	100	100	63 ± 1.5	63 ± 2.3	60 ± 1.3
	25	96 ± 0.6	91 ± 2.3	100	79 ± 4.2	92 ± 1.1	96 ± 2.5	100	100	100	100	100	95 ± 1.3
	250	100	100	100	100	100	100	100	100	100	100	100	100
Tebuconazole	3.2	86 ± 1.7	84 ± 4.7	89 ± 1.1	79 ± 4.2	83 ± 3.3	94 ± 1.4	98 ± 1.0	100	91 ± 1.8	72 ± 1.5	84 ± 6.4	68 ± 5.6
	32	88 ± 1.0	83 ± 3.5	100	100	100	100	100	100	100	100	95 ± 1.1	100
	320	100	100	100	100	100	100	100	100	100	100	100	100
Difenoconazole	2.5	80 ± 2.9	71 ± 2.6	72 ± 2.6	58 ± 3.0	83 ± 5.5	75 ± 2.9	69 ± 2.4	82 ± 2.5	90 ± 1.3	82 ± 2.5	72 ± 1.5	64 ± 2.9
	25	87 ± 1.7	82 ± 1.3	81 ± 1.0	87 ± 2.6	95 ± 2.6	93 ± 1.7	100	90 ± 1.3	96 ± 2.2	100	100	100
	250	97 ± 0.8	90 ± 2.6	96 ± 1.0	94 ± 1.5	94 ± 1.5	98 ± 1.7	100	100	100	100	100	100
Fludioxonil	0.5	100	100	100	33 ± 2.7	11 ± 3.2	4 ± 1.3	80 ± 1.0	69 ± 2.2	79 ± 0.0	80 ± 4.1	83 ± 1.9	72 ± 8.1
	5	100	100	100	36 ± 1.6	23 ± 2.1	19 ± 1.3	70 ± 1.0	70 ± 1.3	72 ± 2.0	80 ± 2.0	89 ± 2.9	77 ± 3.8
	50	100	100	100	46 ± 1.6	39 ± 2.4	40 ± 2.6	74 ± 2.8	70 ± 3.4	80 ± 1.2	84 ± 2.0	90 ± 1.9	83 ± 2.5
Boscalid	5	0	15 ± 0.0	0	11 ± 1.1	12 ± 2.6	0	6 ± 1.2	0	10 ± 1.1	100	100	100
	50	0	11 ± 4.9	0	11 ± 1.1	14 ± 1.3	0	6 ± 2.5	1 ± 0.0	16 ± 1.9	100	100	100

	500	0	30 ± 7.5	30 ± 5.8	3 ± 3.0	13 ± 2.3	0	26 ± 2.1	18 ± 2.5	13 ± 1.1	100	100	100
Isopyrazam	2	18 ± 1.2	25 ± 1.7	19 ± 1.0	15 ± 3.5	0	15 ± 1.3	21 ± 4.0	14 ± 1.3	30 ± 2.7	87 ± 2.5	81 ± 1.0	81 ± 1.8
	20	66 ± 1.4	64 ± 3.0	52 ± 1.0	47 ± 2.0	51 ± 1.3	52 ± 3.3	61 ± 1.1	59 ± 2.6	70 ± 1.0	100	100	100
	200	100	100	100	100	100	100	100	100	100	100	100	100
Thiophanate Methyl	15	100	100	60 ± 6.0	100	100	100	100	79 ± 2.6	83 ± 3.1	75 ± 3.5	82 ± 2.2	81 ± 3.8
	150	100	100	100	100	100	100	100	100	100	100	100	100
	1500	100	100	100	100	100	100	100	100	100	100	100	100
Folpet	12	0	19 ± 1.3	0	0	11 ± 4.0	14 ± 5.1	8 ± 2.4	5 ± 2.5	8 ± 6.3	0	7 ± 2.5	6 ± 1.4
	120	67 ± 3.0	73 ± 2.2	55 ± 1.7	48 ± 1.5	48 ± 1.5	68 ± 1.7	52 ± 2.4	60 ± 1.3	58 ± 2.2	65 ± 2.5	93 ± 5.3	84 ± 1.4
	1200	71 ± 2.2	100	73 ± 1.7	49 ± 1.5	56 ± 5.5	80 ± 0.0	69 ± 2.4	82 ± 2.5	75 ± 1.3	100	100	100

Table S2. Colony growth inhibition on PDA amended with three different concentrations of active ingredient (A.i.) for each fungicide, after 5 days of incubation at 25 °C.

**Tested molecules**	**A.i. concentration (mg L^{-1})**	***F. graminearum***			***F. proliferatum***			***F. verticillioides***			***A. flavus***		
		126	***6352***	***6415***	***12072***	***12103***	***16031***	***12052***	***12043***	***12044***	***8111***	***8115***	***8095***
Prothioconazole	2	100	100	100	100	100	100	100	100	100	100	100	100
	20	100	100	100	100	100	100	100	100	100	100	100	100
	200	100	100	100	100	100	100	100	100	100	100	100	100
Prochloraz	4	100	100	100	100	100	100	100	100	100	100	100	100
	40	100	100	100	100	100	100	100	100	100	100	100	100
	400	100	100	100	100	100	100	100	100	100	100	100	100
Metconazole	0.9	94 ± 1.2	98 ± 0.5	99 ± 0.7	88 ± 2.3	86 ± 0.6	93 ± 0.9	95 ± 1.0	98 ± 0.7	93 ± 1.2	63 ± 3.1	65 ± 1.8	48 ± 2.6
	9	100	100	100	100	100	100	100	100	100	100	100	100
	90	100	100	100	100	100	100	100	100	100	100	100	100
Propiconazole	2.5	59 ± 1.1	76 ± 0.5	80 ± 2.4	59 ± 1.5	73 ± 1.1	73 ± 1.7	85 ± 1.6	95 ± 0.7	88 ± 1.0	66 ± 0.0	65 ± 1.8	52 ± 3.1
	25	89 ± 1.1	91 ± 0.8	97 ± 0.7	72 ± 0.9	88 ± 2.3	87 ± 1.5	98 ± 0.6	100	100	100	90 ± 0.7	90 ± 0.9
	250	97 ± 1.1	100	100	88 ± 1.7	100	100	100	100	100	100	100	100
Tebuconazole	3.2	83 ± 1.5	88 ± 2.2	86 ± 1.1	74 ± 2.6	84 ± 19	88 ± 2.3	95 ± 1.2	98 ± 0.7	99 ± 0.6	96 ± 1.0	75 ± 2.0	65 ± 2.3
	32	85 ± 1.4	91 ± 0.5	100	100	100	100	100	100	100	100	100	100
	320	99 ± 0.7	100	100	100	100	100	100	100	100	100	100	100
Difenoconazole	2.5	79 ± 0.4	76 ± 1.3	75 ± 0.5	70 ± 1.2	74 ± 1.4	80 ± 2.1	86 ± 0.7	87 ± 1.2	87 ± 0.7	59 ± 1.0	64 ± 1.5	60 ± 0.7
	25	81 ± 1.5	77 ± 2.7	80 ± 0.9	84 ± 2.6	90 ± 0.8	88 ± 1.4	100	96 ± 1.2	97 ± 0.7	100	100	88 ± 0.7
	250	92 ± 1.5	91 ± 0.8	92 ± 2.9	89 ± 1.2	91 ± 1.4	100	100	100	100	100	100	100
Fludioxonil	0.5	86 ± 1.1	97 ± 0.4	85 ± 5.5	39 ± 1.8	4 ± 1.4	2 ± 0.8	77 ± 1.2	79 ± 2.1	76.1 ± 4.2	83 ± 1.6	86 ± 1.4	83 ± 1.6
	5	91 ± 6.1	100	83 ± 3.3	29 ± 0.9	10 ± 2.1	18 ± 2.9	68 ± 1.2	65 ± 0.7	61 ± 0.6	83 ± 1.6	88 ± 0.0	81 ± 2.7
	50	94 ± 4.0	100	100.0 ± 0.0	36 ± 1.8	25 ± 0.7	29 ± 2.1	69 ± 0.6	67 ± 0.7	64 ± 1.3	85 ± 1.0	92 ± 1.2	88 ± 1.4
Boscalid	5	0	21 ± 4.9	1 ± 1.3	4 ± 2.0	5 ± 2.1	2 ±0.8	8 ± 2.5	8 ± 2.1	11 ± 2.0	100	100	100
	50	26 ± 2.5	15 ± 2.9	5 ± 0.8	5 ± 0.7	5 ± 1.6	3 ± 0.8	5 ± 2.1	4 ± 2.1	11 ± 2.3	100	100	100
	500	31 ± 2.6	9 ± 4.5	3 ± 1.3	5 ± 2.5	7 ± 2.1	2 ± 2.7	23 ± 3.1	16 ± 1.9	16 ± 1.3	100	100	100
Isopyrazam	2	12 ± 0.7	17 ± 1.9	24 ± 2.8	10 ± 1.4	0	5 ± 0.7	31 ± 0.6	17 ± 0.7	36 ± 1.2	83 ± 0.9	77 ± 1.2	81 ± 1.8
	20	61 ± 2.9	63 ± 0.8	49 ± 0.6	39 ± 1.4	50 ± 0.8	52 ± 6.3	63 ± 0.6	61 ± 0.7	68 ± 0.0	88 ± 0.9	94 ± 0.6	92 ± 0.0
	200	100	100	100	100	100	100	100	100	100	100	100	100
Thiophanate Methyl	15	96 ± 1.4	95 ± 1.8	78 ± 2.9	100	94 ± 1.2	89 ± 2.9	100	86 ± 1.4	92 ± 1.7	85 ± 1.9	87 ± 3.0	85 ± 0.8
	150	100	100	100	100	100	100	100	100	100	100	100	100

	1500	100	100	100	100	100	100	100	100	100	100	100	100
	12	5 ± 0.8	7 ± 2.1	14 ± 2.6	1 ± 0.7	1 ± 0.8	22 ± 7.1	0.	0.	7 ± 2.3	10 ± 1.0	0	9 ± 0.7
Folpet	120	64 ± 2.9	69 ± 0.8	60 ± 0.8	48 ± 1.4	49 ± 0.8	62 ± 0.8	43 ± 1.4	53 ± 0.7	54 ± 0.7	52 ± 0.0	64 ± 1.5	65 ± 0.7
	1200	72 ± 1.1	83 ± 0.5	77 ± 1.7	60 ± 1.9	50 ± 0.8	79 ± 1.6	65 ± 0.0	75 ± 2.4	75 ± 1.8	87 ± 2.6	82 ± 3.2	94 ± 3.1

Table S3. Colony growth inhibition on PDA amended with three different concentrations of active ingredient (A.i.) for each fungicide, after 7 days of incubation at 25 °C.

Tested molecules	A.i. concentration (mg L^{-1})	*F. graminearum*			*F. proliferatum*			*F. verticillioides*			*A. flavus*		
		126	***6352***	***6415***	***12072***	***12103***	***16031***	***12052***	***12043***	***12044***	***8111***	***8115***	***8095***
Prothioconazole	2	100	100	100	100	100	100	100	100	100	100	100	100
	20	100	100	100	100	100	100	100	100	100	100	100	100
	200	100	100	100	100	100	100	100	100	100	100	100	100
Prochloraz	4	100	100	100	100	100	96 ± 1.0	100	100	100	100	100	100
	40	100	100	100	100	100	100	100	100	100	100	100	100
	400	100	100	100	100	100	100	100	100	100	100	100	100
Metconazole	0.9	>88 ± 1.9	>91 ± 0.4	96 ± 1.0	86 ± 0.6	86 ± 1.6	83 ± 1.6	95 ± 0.8	97 ± 0.5	91 ± 0.4	55 ± 0.9	55 ± 1.3	47 ± 2.0
	9	100	100	100	100	100	100	100	100	100	100	100	93 ± 0.6
	90	100	100	100	100	100	100	100	100	100	100	100	100
Propiconazole	2.5	>43 ± 1.0	>70 ± 0.7	67 ± 1.8	56 ± 1.8	75 ± 0.5	73 ± 1.2	84 ± 1.2	94 ± 1.4	85 ± 0.7	56 ± 0.9	62 ± 0.0	56 ± 0.0
	25	>82 ± 1.7	>90 ± 0.7	93 ± 0.5	73 ± 1.0	88 ± 2.3	88 ± 1.8	95 ± 1.1	100	100	100	88 ± 1.0	84 ± 1.5
	250	>96 ± 0.7	>98 ± 0.6	100	90 ± 1.6	100	100	100	100	100	100	100	100
Tebuconazole	3.2	>72 ± 1.7	>86 ± 2.3	82 ± 0.5	75 ± 2.7	84 ± 1.6	83 ± 1.8	90 ± 0.8	96 ± 1.3	95 ± 0.4	58 ± 0.9	72 ± 2.2	68 ± 1.0
	32	>83 ± 1.7	>90 ± 0.4	96 ± 0.0	100	100	100	100	100	100	100	91 ± 0.5	100
	320	>96 ± 0.4	100	100	100	100	100	100	100	100	100	100	100
Difenoconazole	2.5	>73 ± 1.5	>66 ± 0.4	>71 ± 0.0	68 ± 1.3	73 ± 1.7	75 ± 1.9	85 ± 0.5	84 ± 1.0	85 ± 1.0	54 ± 0.8	59 ± 0.5	55 ± 1.1
	25	>74 ± 1.7	>70 ± 1.7	>78 ± 0.7	83 ± 1.8	81 ± 0.0	90 ± 0.9	98 ± 0.5	94 ± 0.5	97 ± 0.5	78 ± 1.6	90 ± 0.5	81 ± 0.5
	250	>87 ± 1.6	>87 ± 1.0	>90 ± 2.7	90 ± 0.5	90 ± 1.7	96 ± 1.4	100	100	100	100	100	100
Fludioxonil	0.5	>76 ± 0.6	>93 ± 0.6	78 ± 12.0	42 ± 0.6	0	0	69 ± 1.1	81 ± 1.0	75 ± 0.0	75 ± 2.7	84 ± 2.0	50 ± 10.0
	5	>76 ± 8.2	>96 ± 0.4	58 ± 7.6	26 ± 1.8	11 ± 1.3	16 ± 1.0	57 ± 0.9	61 ± 2.1	58 ± 1.4	77 ± 1.6	90 ± 1.9	65 ± 0.9
	50	>80 ± 1.3	>97 ± 0.6	100	31 ± 1.6	26 ± 0.5	29 ± 1.5	59 ± 0.4	63 ± 2.1	56 ± 0.0	83 ± 1.6	91 ± 0.5	63 ± 4.6
Boscalid	5	0	0	0	1 ± 0.0	0	10 ± 1.5	6 ± 0.8	7 ± 1.3	5 ± 0.8	100	100	100
	50	0	0	0	4 ± 0.0	1 ± 1.1	10 ± 1.5	3 ± 0.0	3 ± 1.3	8 ± 2.0	100	100	100
	500	0	0	0	1 ± 0.0	5 ± 1.1	10 ± 1.1	7 ± 2.6	8 ± 1.8	11 ± 1.2	100	100	100
Isopyrazam	2	0	0	16 ± 1.1	8 ± 1.4	4 ± 1.1	10 ± 0.5	32 ± 1.7	29 ± 1.0	25 ± 1.7	80 ± 1.9	77 ± 0.8	81 ± 0.9
	20	>50 ± 2.4	>53 ± 0.7	>48 ± 1.1	39 ± 0.5	48 ± 0.6	49 ± 4.5	62 ± 0.5	61 ± 0.5	64 ± 0.0	86 ± 0.7	91 ± 0.5	91 ± 0.5
	200	100	100	100	100	100	100	100	100	100	100	100	100
Thiophanate Methyl	15	>92 ±1.0	>95 ± 1.6	81 ± 1.5	90 ± 1.6	100	91 ± 2.4	100	90 ± 1.0	91 ± 1.6	85 ± 1.6	91 ± 2.5	87 ± 1.9
	150	100	100	100	100	100	100	100	100	100	100	100	100
	1500	100	100	100	100	100	100	100	100	100	100	100	100

	12	>13 ± 0.0	0	>16 ± 2.3	6 ± 1.8	0	23 ± 7.3	0	0	1 ± 0.5	17 ± 2.1	0	4 ± 0.5
Folpet	120	>51 ± 1.6	>62 ± 1.1	>5 ± 0.4	46 ± 0.5	48 ± 1.0	71 ± 1.4	35 ± 1.4	50 ± 0.5	50 ± 1.0	49 ± 0.8	54 ± 1.9	59 ± 0.9
	1200	>66 ± 1.3	>79 ± 0.0	>72 ± 0.6	64 ± 1.5	49 ± 4.0	78 ± 1.4	64 ± 1.4	72 ± 1.8	71 ± 1.0	77 ± 3.4	73 ± 1.9	81 ± 0.5

Table S4. Colony growth inhibition on PDA amended with three different concentrations of active ingredient (A.i.) for each fungicide, after 10 days of incubation at 25 °C.

**Tested molecules**	**A.i. concentration (mg L^{-1})**	***F. graminearum***			***F. proliferatum***			***F. verticillioides***			***A. flavus***		
		126	***6352***	***6415***	***12072***	***12103***	***16031***	***12052***	***12043***	***12044***	***8111***	***8115***	***8095***
Prothioconazole	2	100	100	100	100	100	100	100	100	100	100	100	100
	20	100	100	100	100	100	100	100	100	100	100	100	100
	200	100	100	100	100	100	100	100	100	100	100	100	100
Prochloraz	4	100	100	100	95 ±1.4	>84 ± 1.3	89 ±1.1	100	100	100	100	100	100
	40	100	100	100	100	100	100	100	100	100	100	100	100
	400	100	100	100	100	100	100	100	100	100	100	100	100
Metconazole	0.9	>77 ± 0.7	>86 ± 1.7	90 ± 0.4	85 ± 0.7	>82 ± 1.7	81 ± 1.9	96 ± 0.4	95 ± 0.4	>87 ± 0.7	57 ± 1.1	>52 ± 1.6	52 ± 1.8
	9	100	100	100	100	100	100	100	100	100	100	100	90 ± 1.1
	90	100	100	100	100	100	100	100	100	100	100	100	100
Propiconazole	2.5	>15 ± 0.7	>50 ± 1.1	58 ± 1.5	54 ± 1.1	>67 ± 1.0	70 ± 1.1	91 ± 1.0	90 ± 1.1	>78 ± 1.0	61 ± 2.6	>59 ± 0.4	62 ± 1.3
	25	>63 ± 3.2	>83 ± 0.6	89 ± 1.1	73 ± 0.4	>86 ± 2.0	88 ± 0.8	100	100	>98 ± 0.4	78 ± 2.3	>84 ± 1.5	81 ± 0.7
	250	>96 ± 0.4	>94 ± 1.3	99 ± 0.7	87 ± 1.5	>98 ± 0.4	100	100	100	100	100	100	100
Tebuconazole	3.2	>47 ± 1.3	>80 ± 1.6	74 ± 1.3	73 ± 1.5	>82 ± 0.4	77 ± 1.5	96 ± 0.4	96 ± 0.4	>90 ± 1.3	56 ± 0.0	>71 ± 0.4	66 ± 0.7
	32	>76 ± 2.3	>87 ± 1.1	95 ± 0.4	87 ± 1.5	100	100	100	100	100	100	>84 ± 1.0	93 ± 1.1
	320	>93 ± 0.4	>98 ± 0.4	100	94 ± 1.1	100	100	100	100	100	100	100	100
Difenoconazole	2.5	>67 ± 1.3	>47 ± 1.3	>66 ± 0.7	63 ± 1.1	66 ± 1.7	73 ± 1.6	83 ± 0.9	83 ± 0.9	81 ± 1.2	48 ± 1.4	57 ± 1.6	52 ± 1.6
	25	>66 ± 1.6	>50 ± 1.3	>63 ± 1.3	80 ± 2.6	76 ± 0.5	89 ± 0.8	91 ± 0.5	91 ± 0.4	93 ± 0.8	68 ± 1.4	84 ± 0.8	76 ± 1.4
	250	>83 ± 2.0	>79 ± 1.9	>70 ± 0.7	88 ± 0.9	87 ± 1.3	96 ± 0.9	100	100	98 ± 0.4	100	100	100
Fludioxonil	0.5	>37 ± 3.7	>93 ± 1.0	>70 ± 19.1	47 ± 0.8	>10 ± 0.0	0.0 ± 0.0	>73 ± 1.6	75 ± 1.3	68 ± 1.2	54.5 ± 1.8	75.3 ± 1.2	23.6 ± 7.6
	5	>49 ± 8.3	>94 ± 0.4	>32 ± 9.4	22 ± 1.8	>12 ± 1.0	10 ± 0.4	>48 ± 0.4	58 ± 2.1	45 ± 3.0	54.5 ± 1.0	86.4 ± 0.0	46.1 ± 1.4
	50	>54 ± 0.4	>94 ± 0.6	>80 ± 14.6	28 ± 0.4	>18 ± 0.7	15 ± 1.8	>45 ± 2.6	50 ± 0.4	37 ± 0.0	72.1 ± 1.2	90.1 ± 2.5	45.0 ± 1.3
Boscalid	5	0	0	0	0	0	2 ± 0.4	2 ± 1.1	0	3 ± 1.9	100	100	100
	50	0	0	0	0	0	3 ± 0.0	2 ± 1.4	0	1 ± 0.4	100	100	100
	500	0	0	0	2 ± 1.2	0	4 ± 1.3	8 ± 1.5	9 ± 1.2	4 ± 1.2	100	100	100
Isopyrazam	2	0	0	0	2 ± 0.8	>4 ± 0.9	9 ± 1.1	>13 ± 0.8	9 ± 0.7	10 ± 1.2	81 ± 2.0	77 ± 1.1	81 ± 1.3
	20	>24 ± 2.1	>26 ± 1.0	>36 ± 1.0	31 ± 0.7	>44 ± 0.4	40 ± 1.9	>47 ± 1.6	49 ± 0.4	46 ± 0.8	88 ± 1.0	88 ± 1.5	89 ± 0.9
	200	>95 ± 5.2	>96 ± 4.4	>88 ± 2.3	98 ± 1.1	100	100	100	100	100	100	100	100
Thiophanate	15	>92 ± 1.0	>94 ± 1.9	>84 ± 2.3	93 ± 1.1	>97 ± 1.1	92 ± 2.6	100.0 ± 0.0	92 ± 0.8	91 ± 0.7	79 ± 1.2	88 ± 3.7	85 ± 1.9

Methyl	150	100	100	100	98 ± 2.1	100	100	100	100	100	100	100	100
	1500	100	100	100	100	100	100	100	100	100	100	100	100
Folpet	12	0.0 ± 0.0	0.0 ± 0.0	>14 ± 2.7	5 ± 0.0	0.0 ± 0.0	20 ± 5.1	0.0 ± 0.0	0.0 ± 0.0	4 ± 0.9	0.0 ± 0.0	3 ± 0.9	0.0 ± 0.0
	120	>35 ± 2.4	>48 ± 0.6	>43 ± 0.6	36 ± 0.4	44 ± 1.3	69 ± 0.9	44.1 ± 0.5	44 ± 0.4	43 ± 0.8	43 ± 1.8	53 ± 0.8	45 ± 1.2
	1200	>55 ± 0.7	>71 ± 0.0	>66 ± 0.7	61 ± 1.1	47 ± 1.3	76 ± 0.5	70.3 ± 1.6	70 ± 1.6	70 ± 1.2	63 ± 1.2	78 ± 0.4	68 ± 2.0

Table S5. Conidial germination inhibition on water agar amended with three different concentrations of active ingredient (A.i.) for each fungicide, after 48 hours of incubation at 25 °C.

Tested molecules	A.i. concentration (mg L^{-1})	*F. graminearum*			*F. proliferatum*			*F. verticillioides*			*A. flavus*		
		126	*6352*	*6415*	*12072*	*12103*	*16031*	*12052*	*12043*	*12044*	*8111*	*8115*	*8095*
Prothioconazole	2	-	0	1 ± 0.7	1 ± 0.3	3 ± 0.7	4 ± 0.6	0	1 ± 0.9	2 ± 0.6	100	100	100
	20	-	54 ± 2.8	1 ± 1.0	4 ± 0.9	4 ± 1.0	4 ± 1.3	0	2 ± 0.9	2 ± 0.9	100	100	100
	200	-	72 ± 2.8	2 ± 0.7	6 ± 1.5	6 ± 0.9	4 ± 0.9	0	2 ± 0.7	5 ± 0.6	100	100	100
Prochloraz	4	-	6 ± 2.0	1 ± 0.6	82 ± 1.8	78 ± 1.6	80 ± 1.2	72 ± 1.5	78 ± 1.2	58 ± 1.2	100	100	100
	40	-	7 ± 1.6	2 ± 1.2	100	89 ± 2.6	92 ± 0.9	90 ± 0.9	90 ± 1.2	82 ± 1.2	100	100	100
	400	-	11 ± 2.6	3 ± 1.2	100	99 ± 1.6	93 ± 0.9	99 ± 0.3	99 ± 0.9	96 ± 1.5	100	100	100
Metconazole	0.9	-	88 ± 0.6	1 ± 0.9	87 ± 0.9	19 ± 1.2	85 ± 1.5	88 ±0.4	81 ±1.2	76 ± 1.0	100	100	100
	9	-	93 ± 1.8	3 ± 0.7	93 ± 0.9	89 ± 0.9	90 ± 1.5	91 ±1.6	87 ±1.9	96 ± 1.2	100	100	100
	90	-	100	100	100	100	100	100	100	100	100	100	100
Propiconazole	2.5	-	3 ± 2.7	2 ± 0.9	0	0	4 ± 0.9	41 ± 0.9	22 ± 1.2	33 ± 1.2	34 ± 1.5	44 ± 1.5	50 ± 1.3
	25	-	14 ± 2.1	3 ± 1.8	0	0	55 ± 1.2	75 ± 1.0	59 ± 0.9	61 ± 0.9	100	100	100
	250	-	29 ± 4.4	29 ± 2.5	46 ± 1.5	64 ± 0.7	92 ± 0.9	100	99 ± 0.9	90 ± 0.7	100	100	100
Tebuconazole	3.2	-	9 ± 3.3	2 ± 1.2	30 ± 0.9	0.0 ± 0.0	76 ± 0.9	73 ± 1.5	35 ± 1.5	24 ± 1.2	62.7 ± 1.5	58.5 ± 1.5	54.9 ± 1.6
	32	-	22 ± 2.07	10 ± 1.5	92 ± 1.2	82 ± 1.8	96 ± 1.2	86 ± 0.7	88 ± 1.2	84 ± 0.9	100	100	100
	320	-	100	100	100	100	100	98 ± 0.9	96 ± 1.5	93 ± 0.9	100	100	100
Difenoconazole	2.5	-	4 ± 1.5	3 ± 0.3	2 ± 0.7	3 ± 0.6	37 ± 1.2	15 ± 1.5	6 ± 0.7	6 ± 0.6	100	100	100
	25	-	7 ± 0.9	6 ± 0.9	22 ± 0.9	24 ± 1.2	81 ± 0.9	27 ± 0.9	9 ± 0.3	10 ± 1.5	100	100	100
	250	-	9 ± 0.7	8 ± 1.2	39 ± 0.9	58 ± 0.9	95 ± 1.2	45 ± 0.9	21 ± 1.5	15 ± 1.5	100	100	100
Fludioxonil	0.5	-	8 ± 2.7	0.0 ± 0.0	0	3 ± 1.0	2 ± 0.6	3 ± 0.7	2 ± 1.2	1 ± 0.3	9 ± 1.9	5 ± 1.2	2 ± 0.7
	5	-	15 ± 2.1	0.0 ± 0.0	3 ± 0.3	1 ± 1.2	4 ± 1.5	4 ± 0.6	3 ± 0.6	4 ± 0.9	59 ± 1.5	54 ± 0.3	89 ± 1.3
	50	-	12 ± 2.7	1 ± 0.9	0	2 ± 1.0	7 ± 1.2	4 ± 1.5	3 ± 0.7	5 ± 0.6	99 ± 0.6	88 ± 1.2	94 ± 0.9
Boscalid	5	-	0	0	0	0	1 ± 0.6	4 ± 0.9	0	3 ± 0.3	100	100	100
	50	-	0	0	0	0	1 ± 0.3	6 ± 1.2	0	4 ± 0.9	100	100	100
	500	-	0	0	0	0	2 ± 0.7	8 ± 0.9	0	4 ± 0.3	100	100	100
Isopyrazam	2	-	5 ± 1.2	4 ± 0.6	4 ± 1.0	3 ± 0.6	4 ± 0.6	3 ± 0.9	6 ± 0.9	4 ± 0.3	27 ± 1.2	37 ± 2.2	39 ± 0.7
	20	-	11 ± 0.6	9 ± 1.2	8 ± 1.0	4 ± 0.7	5 ± 0.9	7 ± 0.9	7 ± 1.2	6 ± 0.9	100.0 ± 0.0	100.0 ± 0.0	100.0 ± 0.0
	200	-	100	100	100	100	100	100	100	100	100.0 ± 0.0	100.0 ± 0.0	100.0 ± 0.0
Thiophanate	15	-	75 ± 1.2	2 ± 1.0	31 ± 0.9	8 ± 1.2	3 ± 0.6	86 ± 1.4	26 ± 1.0	36 ± 1.6	1 ± 0.7	5 ± 1.2	1 ± 0.7

Methyl	150	-	84 ± 1.2	5 ± 1.4	43 ± 1.2	22 ± 0.9	3 ± 0.9	100	100	91 ± 0.7	43 ± 1.2	24 ± 1.2	83 ± 0.9
	1500	-	100	33 ± 1.5	86 ± 0.9	67 ± 1.2	3 ± 0.7	100	100	100	100	100	100
Folpet	12	-	8 ± 1.2	5 ± 0.6	32 ± 1.3	29 ± 1.5	87 ± 1.2	25 ± 0.7	2 ± 0.3	5 ± 1.5	73 ± 0.9	89 ± 1.0	89 ± 1.5
	120	-	100	100	100	100	100	100	100	100	100	100	100
	1200	-	100	100	100	100	100	100	100	100	100	100	100

Table S6. Conidial germination inhibition on water agar amended with three different concentrations of active ingredient (A.i.) for each fungicide, after 72 hours of incubation at 25 °C.

Tested molecules	A.i. concentration (mg L$^{-1)}$	*F. graminearum*			*F. proliferatum*			*F. verticillioides*			*A. flavus*		
		126	*6352*	*6415*	*12072*	*12103*	*16031*	*12052*	*12043*	*12044*	*8111*	*8115*	*8095*
Prothioconazole	2	-	100	100	100	100	100	100	100	100	100	100	100
	20	-	100	100	100	100	100	100	100	100	100	100	100
	200	-	100	100	100	100	100	100	100	100	100	100	100
Prochloraz	4	-	6 ± 2.0	1.0 ± 0.6	82 ± 1.8	100	100	100	100	100	100	100	100
	40	-	100	100	100	100	100	100	100	100	100	100	100
	400	-	100	100	100	100	100	100	100	100	100	100	100
Metconazole	0.9	-	100	100	100	100	100	100	100	100	100	100	100
	9	-	100	100	100	100	100	100	100	100	100	100	100
	90	-	100	100	100	100	100	100	100	100	100	100	100
Propiconazole	2.5	-	3 ± 2.7	2 ± 0.9	0	0	4 ± 0.9	41 ± 0.9	22 ± 1.2	33 ± 1.2	34 ± 1.5	44 ± 1.5	50 ± 1.3
	25	-	100	100	100	100	100	100	100	100	100	100	100
	250	-	100	100	100	100	100	100	100	100	100	100	100
Tebuconazole	3.2	-	9 ± 3.3	2 ± 1.2	30 ± 0.9	0	76 ± 0.9	100	100	100	63 ± 1.5	58 ± 1.5	100
	32	-	100	100	100	100	100	100	100	100	100	100	100
	320	-	100	100	100	100	100	100	100	100	100	100	100
Difenoconazole	2.5	-	4 ± 1.5	3 ± 0.3	100	100	100	100	100	100	100	100	100
	25	-	100	100	100	100	100	100	100	100	100	100	100
	250	-	100	100	100	100	100	100	100	100	100	100	100
Fludioxonil	0.5	-	8 ± 2.7	0	0	3 ± 1.0	2 ± 0.6	3 ± 0.7	2 ± 1.2	1 ± 0.3	9 ± 1.9	5 ± 1.2	2 ± 0.7
	5	-	15 ± 2.1	0	3 ± 0.3	1 ± 1.2	4 ± 1.5	4 ± 0.6	3 ± 0.6	4 ± 0.9	59 ± 1.5	54 ± 0.3	90 ± 1.3
	50	-	12 ± 2.7	1 ± 0.9	0	2 ± 1.0	7 ± 1.2	4 ± 1.5	3 ± 0.7	5 ± 0.6	99 ± 0.6	88 ± 1.2	94 ± 0.9
Boscalid	5	-	0	0	0	0	1 ± 0.6	4 ± 0.9	0	3 ± 0.3	100	100	100
	50	-	0	0	0	0	1 ± 0.3	6 ± 1.2	0	4 ± 0.9	100	100	100
	500	-	0	0	0	0	2 ± 0.7	8 ± 0.9	0	4 ± 0.3	100	100	100
Isopyrazam	2	-	5 ± 1.2	4 ± 0.6	4 ± 1.0	3 ± 0.6	4 ± 0.6	3 ± 0.9	6 ± 0.9	4 ± 0.3	27 ± 1.2	37 ± 2.2	39 ± 0.7
	20	-	11 ± 0.6	9 ± 1.2	8 ± 1.0	4 ± 0.7	5 ± 0.9	7 ± 0.9	7 ± 1.2	6 ± 0.9	100	100	100
	200	-	100	100	100	100	100	100	100	100	100	100	100
Thiophanate Methyl	15	-	75 ± 1.2	2 ± 1.0	31 ± 0.9	100	100	100	100	100	100	100	100
	150	-	100	100	100.0 ± 0.0	100	100	100	100	100	100	100	100
	1500	-	100	100	100.0 ± 0.0	100	100	100	100	100	100	100	100

	12	-	8 ± 1.2	5 ± 0.6	32 ± 1.3	29 ± 1.5	87 ± 1.2	100	100	100	73 ± 0.9	89 ± 1.0	89 ± 1.5
Folpet	120	-	100	100	100	100	100	100	100	100	100	100	100
	1200	-	100	100	100	100	100	100	100	100	100	100	100

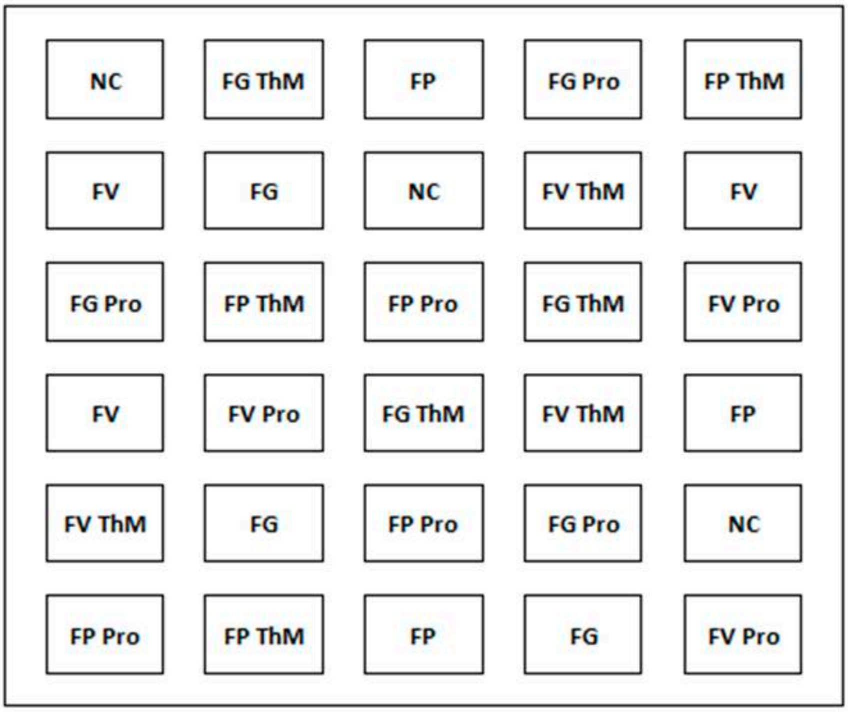

Figure S1. Randomized block experimental design used in the field experiment trial to test the effectiveness of prothioconazole (Pro) and thiophanate-methyl (ThM) treatments compared to untreated theses inoculated with *Fusarium graminearum* (FG), *F. proliferatum* (FP) and *F. verticillioides* (FV) strains. Untreated and not inoculated thesis (NC) was also included.

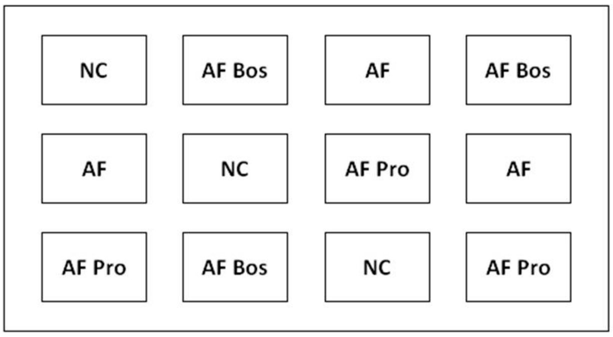

Figure S2. Randomized block experimental design used in the field experiment trial to test the effectiveness of prothioconazole (Pro) and boscalid (Bos) treatments compared to untreated theses inoculated with *Aspergillus flavus* (AF). Untreated and not inoculated thesis (NC) was also included.
